# Fanconi anemia complementation group D2 promotes sensitivity of endometrial cancer cells to chemotherapeutic agents by inhibiting the ferroptosis pathway

**DOI:** 10.1186/s12905-023-02857-4

**Published:** 2024-01-13

**Authors:** Hai-Hong Lin, Wei-Hong Zeng, Hai-Kun Yang, Li-Shan Huang, Ru Pan, Nan-Xiang Lei

**Affiliations:** Department of Gynaecology, Meizhou People’s Hospital, Meizhou Academy of Medical Sciences, Meizhou, Guangdong 514031 China

**Keywords:** Fancd2, Endometrial cancer, Chemotherapeutic agents, Drug resistance, Ferroptosis

## Abstract

**Background:**

Resistance can develop during treatment of advanced endometrial cancer (EC), leading to unsatisfactory results. Fanconi anemia complementation group D2 (Fancd2) has been shown to be closely related to drug resistance in cancer cells. Therefore, this study was designed to explore the correlation of Fancd2 with EC resistance and the mechanism of Fancd2.

**Methods:**

Real-time quantitative PCR (RT-qPCR) was used to detect the expression of Fancd2 in EC tissues and cells. EC cells (Ishikawa) and paclitaxel-resistant EC cells (Ishikawa/TAX) were transfected to knock down Fancd2. In addition, the ferroptosis inhibitor Ferrostatin-1 was adopted to treat Ishikawa/TAX cells. The sensitivity of cancer cells to chemotherapeutic agents was observed via 3-(4,5-dimethyl-2-thiazolyl)-2,5-diphenyl-2-H-tetrazolium bromide (MTT) assay, and inhibitory concentration (IC)50 was calculated. Reactive oxygen species (ROS) levels were measured by flow cytometry, the activity of malondialdehyde (MDA) and the levels of glutathione (GSH) and Fe^2+^ in cells were detected by corresponding kits, and protein expression of solute farrier family 7 member 11 (SLC7A11) and glutathione peroxidase 4 (GPX4) was obtained through western blot.

**Results:**

Compared with the normal tissues and endometrial epithelial cells, Fancd2 expression was significantly increased in EC tissues and Ishikawa cells, respectively. After knock-down of Fancd2, Ishikawa cells showed significantly increased sensitivity to chemotherapeutic agents. Besides, compared with Ishikawa cells, the levels of ROS, the activity of MDA, and the levels of GSH and Fe^2+^ were significantly decreased in Ishikawa/TAX cells, while the expression levels of SLC7A11 and GPX4 were significantly increased. Knock-down of Fancd2 significantly increased the ferroptosis levels in Ishikawa/TAX cells, but this effect could be reversed by Ferrostatin-1.

**Conclusion:**

Fancd2 increases drug resistance in EC cells by inhibiting the cellular ferroptosis pathway.

**Supplementary Information:**

The online version contains supplementary material available at 10.1186/s12905-023-02857-4.

## Introduction

As a common malignancy threatening women's health, endometrial cancer (EC) is characterized with a continuously increasing incidence over the past decade. At present, EC has become the second leading cause of gynecological cancer-related death in women [1]. For a long time, there are no clear standard classification methods for the molecular classification of EC. In 2013, the Cancer Genome Atlas (TCGA) research network broke through the limitations of the CE classification by integrating molecular characterization. However, such classification method is limited by the main disadvantages of its considerable complexity and impracticality in clinical practice [2, 3]. According to the classification by the World Health Organization (WHO) in 2014, EC is divided into atypical hyperplasia and atypical hyperplasia. Among patients with atypical hyperplasia, 32 to 37% of cases may develop EC, with a high risk of progression up to 25% [4]. It was reported that EC patients diagnosed in the early stage have a good prognosis with a 5-year survival rate of ≥ 95%. However, the survival rate is significantly reduced in patients diagnosed with advanced or recurrent EC, and their 5-year survival rate is less than 20% even after combination therapy [5]. Chemotherapy and hormone therapy are the main management strategies for patients with advanced EC. The main reason for unsatisfactory treatment outcomes is the emergence of drug-resistant tumor cells during treatment. Long-term use of chemotherapeutic drugs makes tumor cells that initially respond to anticancer agents become insensitive or even resistant [6]. Doxorubicin, sorafenib, cisplatin and paclitaxel (also known as taxol) all are prevalent drugs for the treatment of EC, and although the mechanisms are different, they can all induce the death of cancer cells. However, tumor cells resistant to most chemotherapeutic agents, including EC, have been found clinically [7]. Previous studies have revealed the effect of salinomycin on mRNA and miRNA expression of drug-resistant genes in Ishikawa EC cell lines by microarray analysis and RT-qPCR. According to the analysis results, the expression of TUFT1, MTMR11 and SLC30A5 differed most significantly; besides, the influence probability between TUFT1 and hsa-miR-3188 (FC + 2.48), mtmr11 and has-miR-16 (FC-1.74), SLC30A5 and hsa-mir-30d (FC-2.01) was the highest. These results indicated changes in mRNA and miRNA activity involved in drug resistance, and these characteristic changes were expected as a result of anticancer therapy [8]. The underlying causes of drug resistance in malignancies are complex. Vermij L et al. demonstrated that resistance to paclitaxel in EC was attributed to the multidrug resistance 1 (MDR-1) gene expressing the P-glycoprotein (P-gp), and point mutations in the tubulin binding sites that interacted with paclitaxel [9]. For improving the treatment of EC, the molecular mechanism of resistance to anticancer agents needs further investigation.

Fanconi anemia complementation group D2 (Fancd2) is a nuclear protein involved in DNA damage repair. Fancd2 was initially found to be an essential protein for the development of Fanconi anemia [10], but subsequent studies have revealed its association with cancer development. For example, Houghtaling et al. showed that mice lacking Fancd2 were prone to cancers, including acute myeloid leukemia and squamous cell carcinoma [11]. Lisa et al. observed that high expression of Fancd2 promoted excessive proliferation and metastasis of esophageal squamous cell carcinoma cells [12]. Sonali et al. suggested a correlation between subcellular localization of Fancd2 and ovarian cancer survival; and Fancd2 localized in the nucleus led to reduced patient survival [13]. Collectively, the role of Fancd2 in cancers is evident. Fancd2, moreover, has recently been reported to be associated with chemoresistance in cancer cells. As early as 2005, a study by Chirnomas D et al. pointed out that inhibition of the Fanconi anemia pathway was effective in restoring cisplatin sensitivity in ovarian and breast tumor cell lines [14]. Alex et al. also proved that reducing Fancd2 expression could not only restore the sensitivity of the human breast epithelial cell line MCF10A to mitomycin C, but also inhibit the repopulation ability of the cancer cells [15]. In addition, the association between Fancd2 and drug resistance has been reported in multiple myeloma, ovarian cancer, non-small cell lung cancer, and head and neck cancer in vitro [15]. Based on previous studies, we attempted to propose new solutions to solve EC drug resistance through exploring the correlation of Fancd2 with EC development and chemoresistance in EC.

## Materials and methods

### Tissue specimens

Tissue specimens were collected from 20 patients pathologically diagnosed as EC in Meizhou People’s Hospital, Meizhou Academy of Medical Sciences between January 2016 and May 2019. These patients did not receive any chemotherapy or radiotherapy before surgery, and tumor tissue (EC group) and adjacent normal tissue (Normal group) were obtained after surgery. The tissue specimen collection was approved by the ethical committee of Meizhou People’s Hospital, Meizhou Academy of Medical Sciences (Ethical No.: 2022-C-83) and written informed consent was acquired from all patients. The clinical information of patients was shown in Table 1.

**Table 1:** The patient's clinical information

Patient information	Tumor (*n* = 20)
Age (year)	
≤ 60	16
> 60	4
Body mass index (BMI, kg/m^2^)	23.55 ± 2.60
International Federation of Gynecology and Obstetrics (FIGO) staging	
Stage I	18
Stage II	0
Stage III	2
Stage IV	0
Histopathological type	
Endometrioid	19
Serous	1
Clear	0
Others	0
Recurrence risk	
Low-risk	2
Moderate-risk	10
High-risk	8
Unknown	0

### Cell culture

Human endometrial epithelial cells (hEECs, XY-XB-1546) and EC cells (Ishikawa, SNL-171) were purchased from the American Type Culture Institute. Cell culture was achieved in a RPMI-1640 medium supplemented with 10% fetal bovine serum (FBS) and 1% penicillin/streptomycin, with incubation conditions of 37 °C and 5% CO_2_. The Ishikawa cells were exposed to different concentrations of paclitaxel to obtain paclitaxel-resistant cells (Ishikawa/TAX).

### Cell transfection

Ishikawa cells were transfected with negative pcDNA3.1 (Vector group), over-expression plasmid pcDNA3.1-Fancd2 (Fancd2 group), negative siRNA (siNC group), and Fancd2 siRNA (si-Fancd2 group) by lipo3000 transfection kit (LMRNA001, Invitrogen, California, USA). Ishikawa/TAX cells were transfected with negative siRNA (siNC group), Fancd2 siRNA (si-Fancd2 group), and treated with Ferrostatin-1 (Fer-1, 20 μM).

### Real-time quantitative PCR (RT-qPCR)

The tissue specimens were added with 1 mL TRizol (12183555, Invitrogen, California, USA), and then subjected to a thorough homogenization in a homogenizer and centrifugation at 12,000 rpm for 10 min. The obtained supernatant was centrifuged with 200 uL chloroform to acquire new supernatant, followed by addition of 500 uL isopropanol and centrifugation to allow precipitation of RNA. The acquired RNA was dissolved with nuclease-free water. The same procedures were followed to extract RNA from the cells. After that, 1 μg RNA was reversely transcribed into cDNA using M-MLV reverse transcriptase (28025013, Invitrogen, California, USA). Subsequently, real-time quantitative PCR (RT-qPCR) assay was performed using the SYBR Green PCR kit (4344463, Invitrogen, California, USA) on the Quant Studio 6 Flex system (Applied Biosystems, USA), and the cycle threshold (Ct) of each gene was recorded. The relative expression of the target gene was calculated using the 2^−ΔΔCt^ method, with glyceraldehyde-3-phosphate dehydrogenase (GAPDH) as the internal reference gene. Real-time PCR cycles included: 95 °C for 10 min, 40 cycles (95 °C for 15 s, 67 °C for 30 s, 72 °C for 30 s), and 72 °C for 5 min. Primers used were shown in Table 2 3-(4,5-dimethyl-2-thiazolyl)-2,5-diphenyl-2-H-tetrazolium bromide (MTT) assay Ishikawa or Ishikawa/TAX cells were seeded in 96-well plates at 5 × 10^3^ cells/well. Upon completion of transfection with/without Fer-1 treatment, the cells were incubated with different concentrations of paclitaxel, cisplatin, doxorubicin, and sorafenib for 24 h. Then, 3-(4,5-dimethyl-2-thiazolyl)-2,5-diphenyl-2-H-tetrazolium bromide (MTT, C0009S, Beyotime Biotechnology, Shanghai, China) solution (20 μL) was added to each well. Following 4 h of incubation, the reaction was stopped with 150 μL DMSO (ST1276, Beyotime Biotechnology, Shanghai, China) and the cells were shaken for 10 min at room temperature. Subsequently, the absorbance was measured at 490 nm by a microplate reader (Model: 550, BIO-RAD, China) and the viability of cells was calculated.

**Table 2:** RT-qPCR primer sequences

Gene	Sequences (5’ to 3’)
Fancd2	F: 5’- TTCCAGGATGCCTTCGTAGTGG
	R: 5’- GCAGGAGGTTTATGGCAATCCC
GAPDH	F: 5’- GTCTCCTCTGACTTCAACAGCG
	R: 5’- ACCACCCTGTTGCTGTAGCCAA

### Detection of reactive oxygen species level

Ishikawa or Ishikawa/TAX cells were seeded in 12-well plates at 5 × 10^5^ cells/well. Firstly, the cells were subject to transfection with/without Fer-1 treatment. Then, 10 μM carboxy-H2DCFDA (C400, ThermoFisher Scientific, California, USA) was added for 30 min of cell incubation at 37 °C in the dark. Subsequently, the cells were washed twice with PBS, resuspended with trypsin, and then collected. Fluorescence values were measured by flow cytometry (emission 495 nm and absorption 525 nm).

### Apoptosis

In strict accordance with the corresponding instructions, Annexin V-FITC (fluorescein Isothiocyanate)/PI (propidium iodide) apoptosis detection kits (APOAF, Sigma-Aldrich, St. Louis, Missouri, USA) were used to detect the apoptosis level of Ishikawa or Ishikawa/TAX cells after different treatments. In short, the cells were washed with PBS, and the cell density was adjusted. Then, the cells were suspended in a 500 μL binding buffer and incubated with 5 μL Annexin V-FITC and 5 μL PI for 30 min at room temperature. After that, the cells were transferred into a flow tube and examined on the Accuri C6 flow cytometer (Tomy Digital Biology, CA, USA).

### Colony formation assay

Ishikawa or Ishikawa/TAX cells were seeded in 6-well plates with 1 × 10^3^ cells per well. After 2 weeks of culture, the cells were fixed with 4% paraformaldehyde and then stained with 0.5% crystal violet. The results were observed with a microscope (Nikon, Japan) and analyzed using the ImageJ software.

### Detection of malondialdehyde, glutathione, and Fe^2+^ levels

Ishikawa or Ishikawa/TAX cells were seeded in 6-well plates at 1 × 10^6^ cells/well. Then, the cells were transfected to knock down or over-express Fancd2 and treated with Fer-1. After that, the cells were washed twice with PBS, followed by cell lysis and centrifugation at 12,000 rpm for 10 min. Next, the supernatant was collected for the detection of malondialdehyde (MDA, S0131S) activity, glutathione (GSH, S0052) level, and Fe^2+^ level (S0116) according to the protocol of corresponding kits (Beyotime Biotechnology, Shanghai, China).

### Western blot

Cells were solubilized in RIPA lysis buffer (P0013B, Beyotime Biotechnology, Shanghai, China), and the supernatant was collected after centrifugation at 12,000 rpm for 20 min. Total protein concentration was detected by BCA assay (P0009, Beyotime Biotechnology, Shanghai, China). Proteins were then separated using 12% SDS-PAGE (P0012A, Beyotime Biotechnology, Shanghai, China), and transferred onto polyvinylidene fluoride membranes (PVDF, 88585, ThermoFisher Scientific, California, USA). Upon completion of blocking step in 5% skimmed milk for 1 h, the membranes were incubated overnight at 4 °C with primary antibodies Fancd2 (1:1000, ab108928; Abcam, Cambridge, UK), solute farrier family 7 member 11 (SLC7A11, 1:1000, ab175186; Abcam, Cambridge, UK), and glutathione peroxidase 4 (GPX4, 1:1000, ab252833; Abcam, Cambridge, UK). On the next day, the membranes were incubated with HRP-conjugated secondary antibodies (1:5000, ab205719; Abcam, Cambridge, UK) for 2 h. After that, the protein bands were visualized using the ECL Western blotting Kit (32109, ThermoFisher Scientific, California, USA). Finally, the relative expression of proteins was calculated using GAPDH as an internal reference.

### Statistical analysis

Data were analyzed by Statistical Package for the Social Sciences version 26.0. Differences between two groups were analyzed using paired t tests, and comparisons among multiple groups were analyzed by one-way analysis of variance and Tukey 's post hoc test. *P* < 0.05 was used as the criterion for a significant difference. All experiments were repeated three times.

## Results

### Fancd2 was up-regulated in endometrial cancer and associated with chemoresistance

**Fig. 1:**
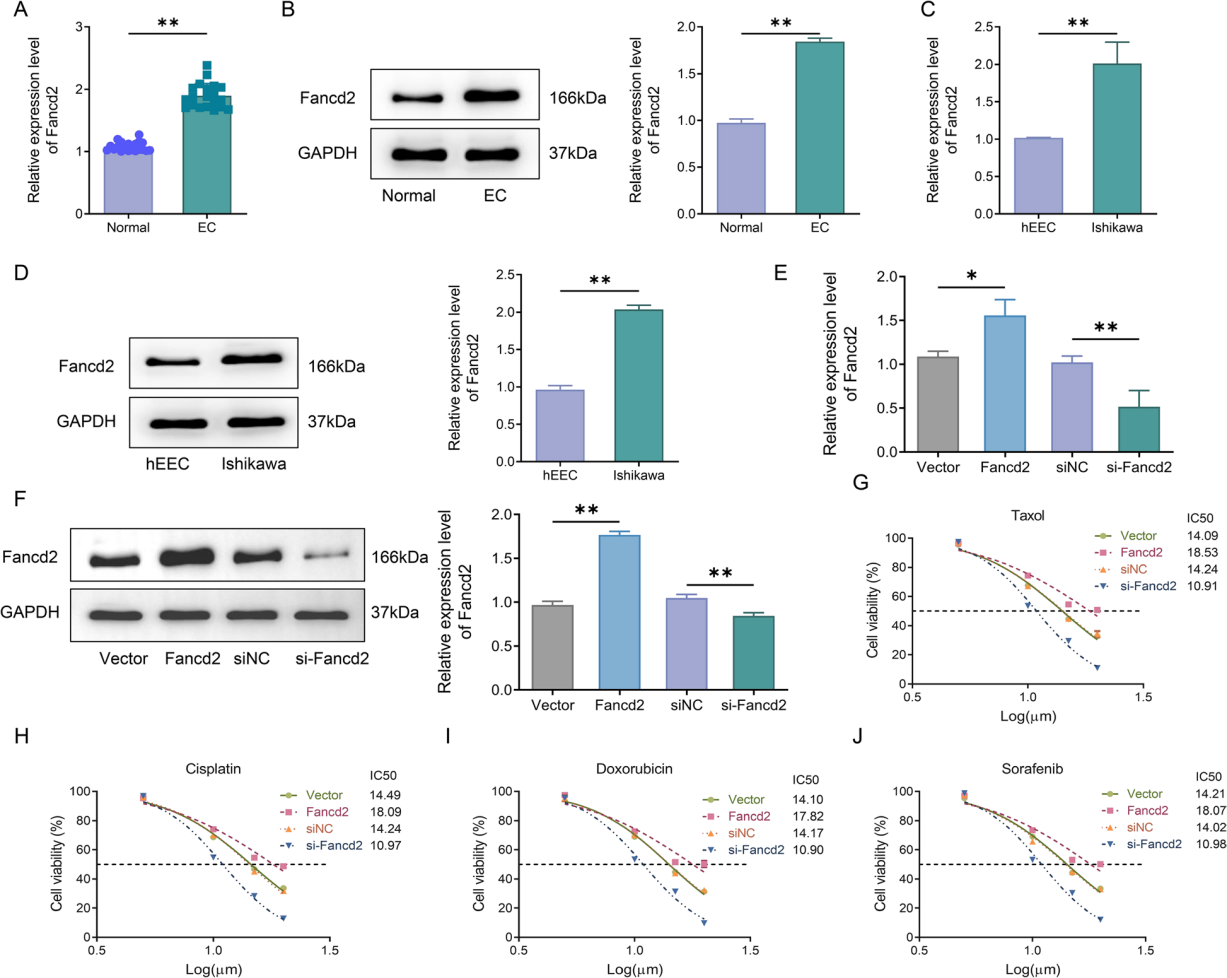
Fancd2 was up-regulated in endometrial cancer and associated with chemoresistance. **A** RT-qPCR was used to detect Fancd2 expression in normal and endometrial cancer (EC) tissues. **B** Western blot was adopted to check the expression level of Fancd2 in the Normal group and EC group. **C** RT-qPCR was employed to detect Fancd2 expression in human endometrial epithelial cells (hEEC) and EC cells (Ishikawa). **D** Western blot was adopted for detecting Fancd2 expression levels in hEEC and Ishikawa cells. **E** RT-qPCR was used for measuring the transfection efficiency of Fancd2 in Ishikawa cells. **F** Western blot was used to examine the expression level of Fancd2 in the Ishikawa cells in Vector group, Fancd2 group, siNC group and si-Fancd2 group. **G**–**J** MTT was utilized to test the cell activity of Ishikawa cells treated with different concentrations of paclitaxel (also known as taxol) (**G**), cisplatin (**H**), doxorubicin (**I**), and sorafenib (**J**). * *P* < 0.05, ** *P* < 0.01

Fancd2 expression in EC tissues and cells was measured to explore the role of Fancd2 in EC. RT-qPCR and western blot showed that Fancd2 expression was significantly increased in the EC group compared with the Normal group (Fig. 1A, B), and was markedly higher in Ishikawa cells than in hEECs (Fig. 1C, D). Further, to observe the effect of Fancd2 on EC chemoresistance, Ishikawa cells were subjected to transfection to inhibit or enhance Fancd2 expression. RT-qPCR and western blot confirmed the up-regulation of Fancd2 in the Fancd2 group compared with the Vector group and the down-regulation of Fancd2 in the si-Fancd2 group compared with the siNC group (Fig. 1E, F). Subsequently, the effect of over-expression or knock-down of Fancd2 on drug resistance in Ishikawa cells was assessed by MTT assay. The assay results demonstrated that Ishikawa cells over-expressing Fancd2 presented with significantly increased inhibitory concentration (IC)50 under paclitaxel, cisplatin, doxorubicin, and sorafenib treatment, while knock-down of Fancd2 showed the opposite outcome (Fig. 1G–J). These results indicated that Fancd2 was up-regulated in EC and was associated with resistance to chemotherapy.

### Fancd2 was up-regulated in Ishikawa/TAX cells and associated with paclitaxel resistance

**Fig. 2:**
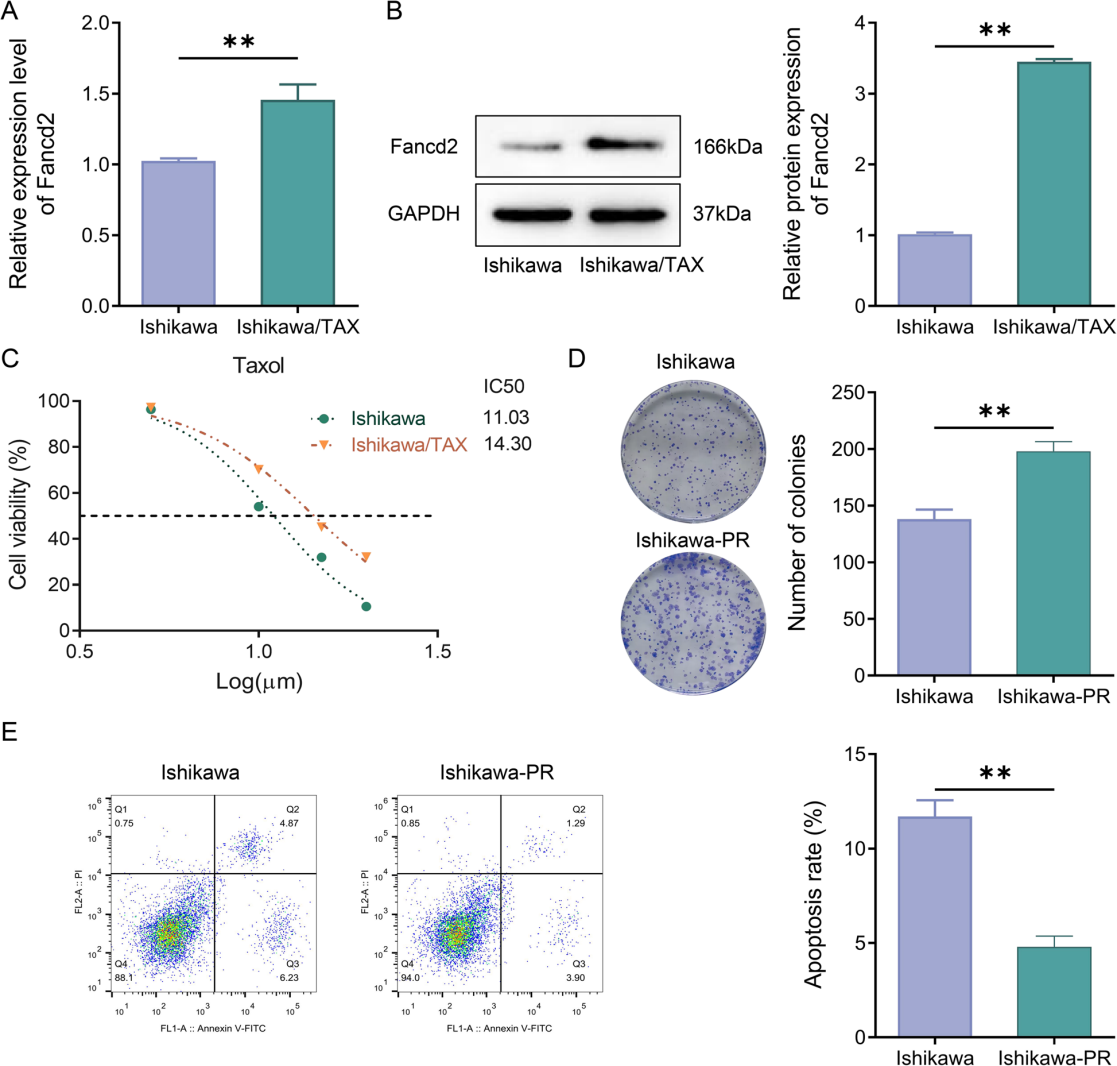
Fancd2 was up-regulated in Ishikawa/TAX cells and associated with paclitaxel resistance. **A** Fancd2 mRNA expression levels in Ishikawa cells and Ishikawa/TAX cells were assessed by RT-qPCR. **B** Western blot was used to detect Fancd2 protein expression levels in Ishikawa cells and Ishikawa/TAX cells. **C** MTT assay was applied to test the cellular activity of Ishikawa cells and Ishikawa/TAX cells under paclitaxel treatment. **D** Clonal formation assay was utilized to determine the number of cell clones in Ishikawa cells and Ishikawa-TAX cells. **E** Flow cytometry was employed to detect apoptosis in Ishikawa cells and Ishikawa-TAX cells. ** *P* < 0.01

Measurement of Fancd2 expression in Ishikawa/TAX cells was conducted for further observing the effect of Fancd2 on chemoresistance in Ishikawa cells. Based on the RT-qPCR and western blot results, the expression of Fancd2 was much higher in Ishikawa/TAX cells than in Ishikawa cells (Fig. 2A, B). Subsequently, MTT assay revealed that compared with Ishikawa cells, IC50 was markedly increased in Ishikawa/TAX cells after paclitaxel treatment (Fig. 2C). Clonal formation experiment exhibited that the number of cell clones in Ishikawa-PR cells increased significantly compared with Ishikawa cells (Fig. 2D). Besides, flow cytometry further revealed that compared with Ishikawa cells, the level of apoptosis in Ishikawa PR cells was significantly reduced (Fig. 2E). These results suggested that up-regulation of Fancd2 expression was possibly associated with cellular resistance to paclitaxel.

### Ferroptosis was decreased in Ishikawa/TAX cells

**Fig. 3:**
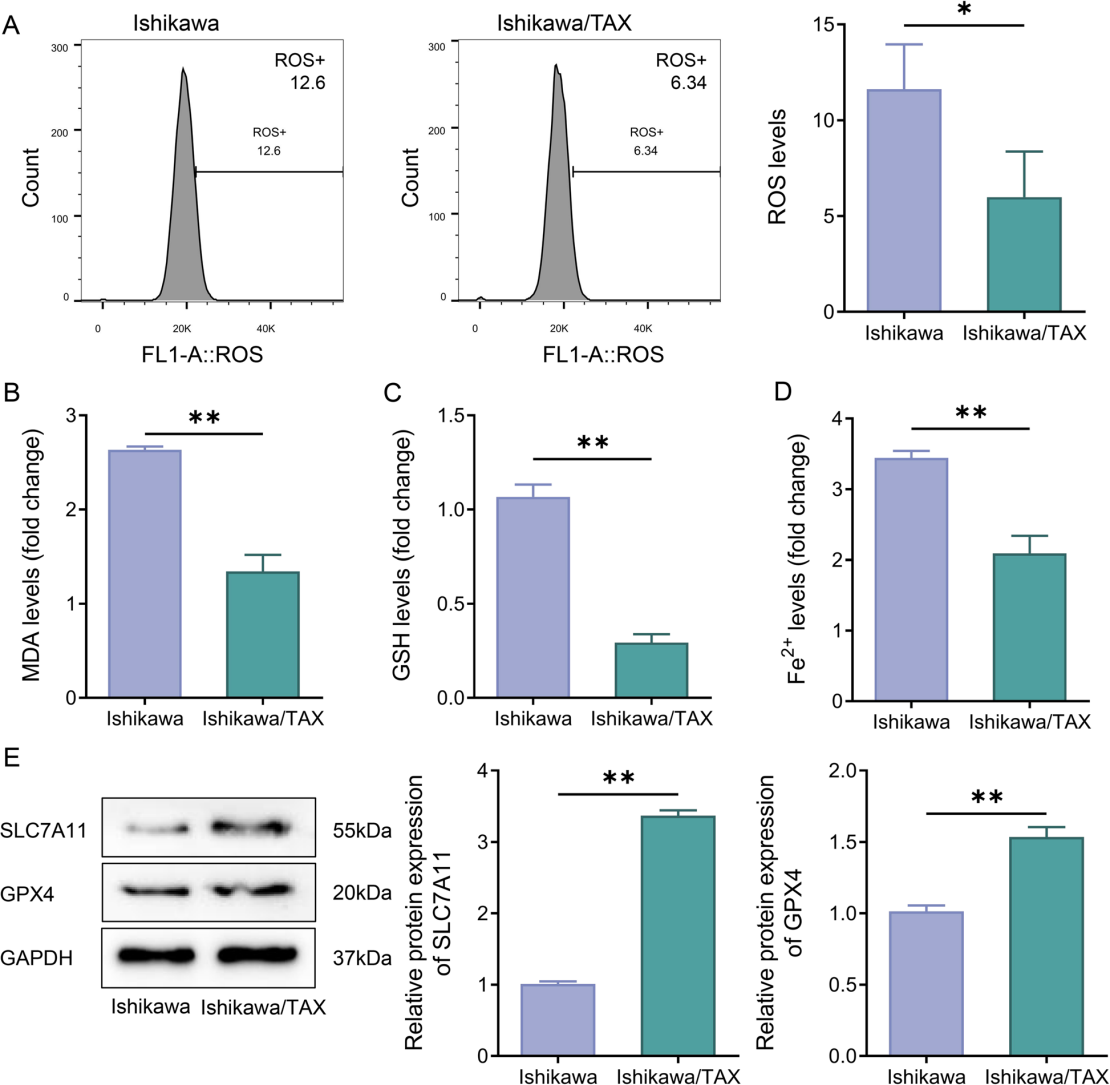
Ferroptosis was decreased in Ishikawa/TAX cells. **A** The level of reactive oxygen species (ROS) level in Ishikawa cells and Ishikawa/TAX cells was detected by flow cytometry. **B**–**D** Malondialdehyde (MDA) activity (**B**), glutathione (GSH) level (**C**), and Fe^2+^ (**D**) in Ishikawa cells and Ishikawa/TAX cells. **E** The protein expression levels of SLC7A11 and GPX4 in Ishikawa cells and Ishikawa/TAX cells were detected by western blot. *P < 0.05, ** *P* < 0.01

Ferroptosis is a common mode of death in cancer cells [16]. Detection of ROS level, MDA activity, GSH, and Fe^2+^ levels in Ishikawa/TAX cells allowed the determination of ferroptosis level in cells. Compared with Ishikawa cells, Ishikawa/TAX cells showed a significant decline in ROS level, MDA activity, GSH, and Fe^2+^ levels (Fig. 3A–D). Subsequently, the protein expression levels of SLC7A11 and GPX4 in the cells were detected by western blot. The western blot outcomes showed that the protein expression levels of SLC7A11 and GPX4 in the Ishikawa/TAX group were significantly higher than those in the Ishikawa group (Fig. 3E). The above results indicated a decrease in ferroptosis levels in paclitaxel-resistant Ishikawa cells.

### Knock-down of Fancd2 improved paclitaxel sensitivity by promoting ferroptosis

**Fig. 4:**
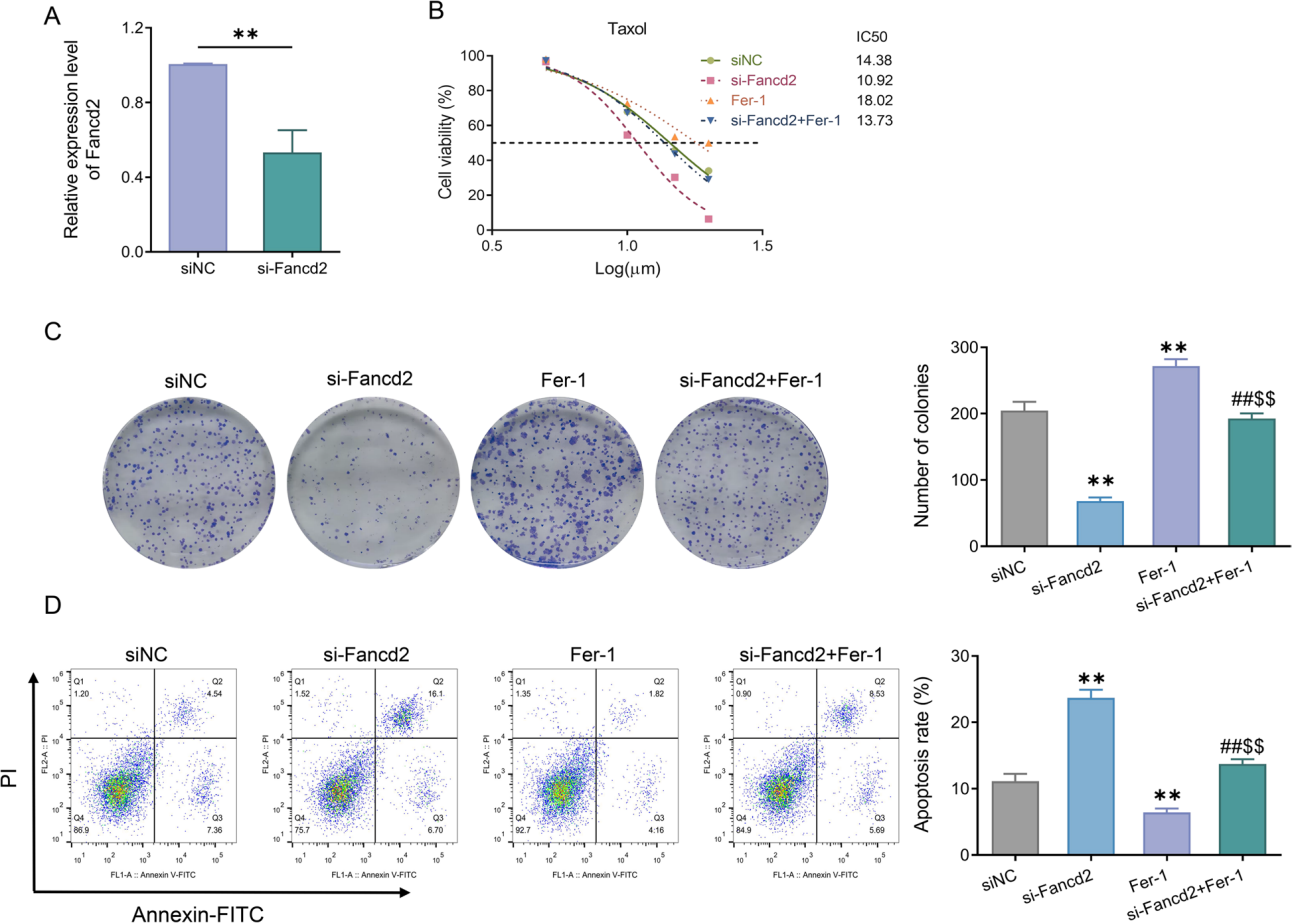
Knock-down of Fancd2 promoted ferroptosis in Ishikawa/TAX cells. **A** RT-qPCR was used to detect the expression level of Fancd2 in Ishikawa/TAX cells. **B** MTT assay was adopted to assess the cell viability of Ishikawa/TAX cells under paclitaxel treatment, and IC50 was calculated. **C** Clonal formation assay was employed to examine the number of cell clones in the siNC group, si-Fancd2 group, Fer-1 group and si-Fancd2 + Fer-1 group. **D** Flow cytometry was utilized for evaluating the apoptosis level in the siNC group, si-Fancd2 group, Fer-1 group, and si-Fancd2 + Fer-1 group. ** *P* < 0.01 *vs*. siNC; ## *P* < 0.01 *vs*. si-Fancd2; $$ *P* < 0.01, *vs*. Fer-1

Subsequently, Fancd2 expression was knocked down by transfection of Fancd2 siRNA into Ishikawa/TAX cells. RT-qPCR showed that Fancd2 expression levels were significantly reduced in Ishikawa/TAX cells in the si-Fancd2 group compared with the siNC group (Fig. 4A). Additionally, MTT assay results indicated that the IC50 of cells in the si-Fancd2 group was significantly lower than that in the siNC group (Fig. 4B). Furthermore, the clone formation experiment showed that compared with the siNC group, the number of cell clones in the si-Fancd2 group significantly decreased, while the number of cell clones in the Fer-1 group significantly increased; relative to the si-Fancd2 group, the number of cell clones in the si-Fancd2 + Fer-1 group significantly increased; in contrast to the Fer-1 group, the number of cell clones in the si-Fancd2 + Fer-1 group was significantly reduced (Fig. 4C). Flow cytometry showed that compared with the siNC group, the apoptosis level in the si-Fancd2 group was significantly increased, while that in the Fer-1 group was significantly decreased. In comparison with the si-Fancd2 group, the level of apoptosis in the si-Fancd2 + Fer-1 group was significantly reduced; while compared with the Fer-1 group, the apoptosis level in the si-Fancd2 + Fer-1 group was significantly increased (Fig. 4D).

**Fig. 5:**
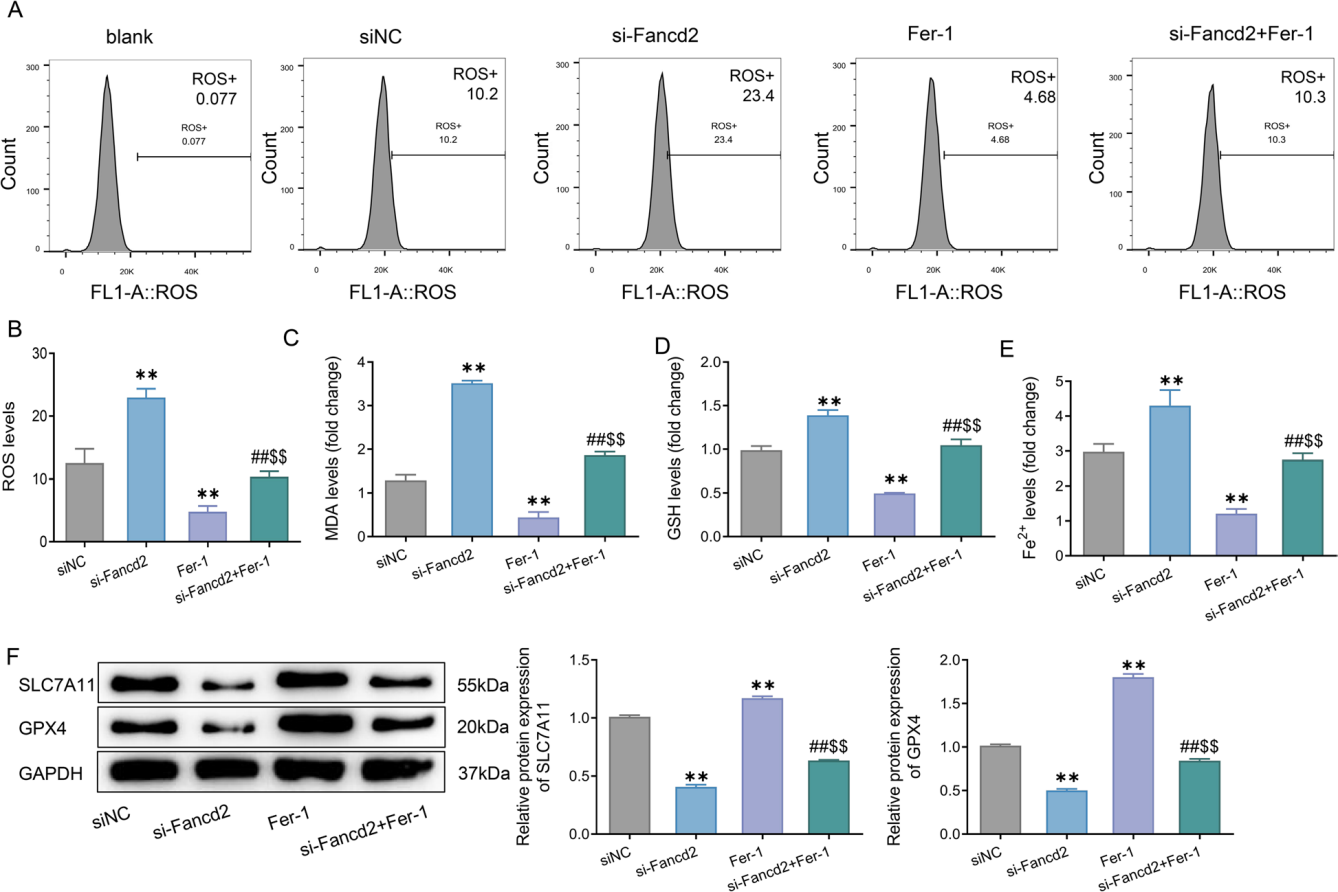
Knock-down of Fancd2 increased the sensitivity of Ishikawa/TAX cells to paclitaxel by promoting ferroptosis. **A**, **B** Flow cytometry was adopted to detect the level of reactive oxygen species (ROS) in Ishikawa/TAX cells. **C**–B Malondialdehyde (MDA) activity (**C**), glutathione (GSH) level (**D**), and Fe^2+^ (**E**) in Ishikawa/TAX cells. **F** The protein expression levels of SLC7A11 and GPX4 in Ishikawa/TAX cells were assessed via western blot. ** *P* < 0.01 *vs*. siNC; ## *P* < 0.01 *vs*. si-Fancd2; $$ *P* < 0.01, *vs*. Fer-1

To verify the relationship of Fancd2 over-expression with cellular resistance and ferroptosis, Fancd2 was knocked down, and the ferroptosis inhibitor Fer-1 was employed to treat cells. The results showed that knock-down of Fancd2 significantly increased the levels of ROS, GSH, and Fe^2+^ and the activity of MDA in Ishikawa/TAX cells compared with the siNC group (Fig. 5A–E). Western blot results also revealed that knock-down of Fancd2 significantly reduced the protein expression levels of SLC7A11 and GPX4 in Ishikawa/TAX cells (Fig. 5F). However, the effect of Fancd2 knock-down on ferroptosis in Ishikawa/TAX cells could be significantly inhibited after Fer-1 treatment. Collectively, knock-down of Fancd2 improved the sensitivity of Ishikawa/TAX cells to paclitaxel by inducing ferroptosis.

## Discussion

Although treatment strategies for EC have gradually improved, patients with advanced EC are prone to develop drug resistance during chemotherapy, seriously affecting the therapeutic effect [17]. Several recent studies have revealed genes associated with chemosensitivity, and proposed some novel and promising approaches to address resistance [18]. In this study, Fancd2 expression was up-regulated in EC tissues, and more importantly, Fancd2 was associated with sensitivity to chemotherapeutic agents in EC.

Previous studies have linked Fancd2 to susceptibility to cancer. Fancd2 is a key player in the DNA repair pathway and is important for maintaining genomic stability in response to various gene damage [13]. Recently, there are studies that up-regulation of Fancd2 expression is positively correlated with tumor size and poor prognosis in ovarian cancer, nasopharyngeal carcinoma, glioblastoma, and EC [19]. Consistent with previous reports, we observed a significant up-regulation of Fancd2 expression in EC tissues and EC cell lines (Ishikawa); and interestingly, after knock-down of Fancd2 expression in Ishikawa, the cells showed a marked decrease in resistance to the above-mentioned chemotherapeutic agents. Yao C et al. demonstrated that Fancd2 was associated with doxorubicin resistance in leukemia [20]. Dai et al. found that cisplatin resistance in drug-resistant lung cancer cells could be effectively reversed by inhibiting the gene expression level of the Fancd2/BRCA pathway [21]. In addition, the results of this study showed that Fancd2 expression was significantly increased in Ishikawa/TAX cells compared with Ishikawa cells, and knocking down Fancd2 could restore the sensitivity of Ishikawa/TAX cells to paclitaxel. Also Xiao et al. reported that curcumin reversed the multidrug resistance of multiple myeloma cells MOLP-2/R by inhibiting the Fancd2 pathway [22]. Taken together, these findings suggest that high expression of Fancd2 may be associated with drug resistance.

In the course of exploring the mechanism of Fancd2 in EC, we observed that Fancd2 expression was associated with ferroptosis. Different from apoptosis, necrosis, and autophagy, ferroptosis is an intracellular iron-dependent form of cell death [23]. Briefly, ferroptosis is characterized by an imbalance in the redox state and manifested as an increase in the ROS level [24]. Friedmann A J et al. pointed out that tumor cells could significantly enhance their defense against oxidative stress by negatively regulating ferroptosis [25]. In this study, we found a significant decline in ROS level, MDA activity, GSH level and Fe^2+^ level, and a marked increase in SLC7A11 and GPX4 expression in Ishikawa/TAX cells. Such results indicated a low level of ferroptosis in Ishikawa/TAX cells. After knock-down of Fancd2 expression, the above-mentioned ferroptosis-related indicators showed a significant opposite change, indicating a marked increase in ferroptosis levels. Zhang C et al. revealed that Fancd2 was a protein associated with ferroptosis, and high levels of Fancd2 significantly inhibited cellular ferroptosis levels [23]. Song et al. demonstrated that abnormal expression of Fancd2 led to ferroptosis and was associated with temozolomide resistance in glioblastoma [26]. These findings yield a conclusion that knock-down of Fancd2 in Ishikawa/TAX cells induces cellular ferroptosis and increases drug sensitivity.

This study preliminarily demonstrated in vitro that chemotherapy resistance of Fancd2 in Ishikawa cells was closely related to the reduction of ferroptosis levels. However, there are many shortcomings in this study. First, the results of this study were not further verified through animal experiments. Second, in vitro studies, we used only one cell line and did not perform similar experiments in multiple cell lines and corresponding cell lines of the G2 and G3 phases of the EC. These defects need to be further discussed in the future research.

## Conclusion

Fancd2 expression was significantly up-regulated in EC. Besides, Fancd2 led to chemoresistance by decreasing ferroptosis levels in Ishikawa EC cell lines. Therefore, Fancd2 may serve as a biomarker and therapeutic target for chemoresistance in EC. This study provides a new approach to address multi-drug resistance in EC cells.

### Supplementary Information

**Additional file 1.**

## Data Availability

The authors confirm that the data supporting the findings of this research are available within the article.
