# Supplementary material for: Fanconi anemia complementation group D2 promotes sensitivity of endometrial cancer cells to chemotherapeutic agents by inhibiting the ferroptosis pathway
Source: BMC Womens Health. 2024 Jan 13;24:41. doi: 10.1186/s12905-023-02857-4 (PMC10787983; doi:10.1186/s12905-023-02857-4)

Figure 1B

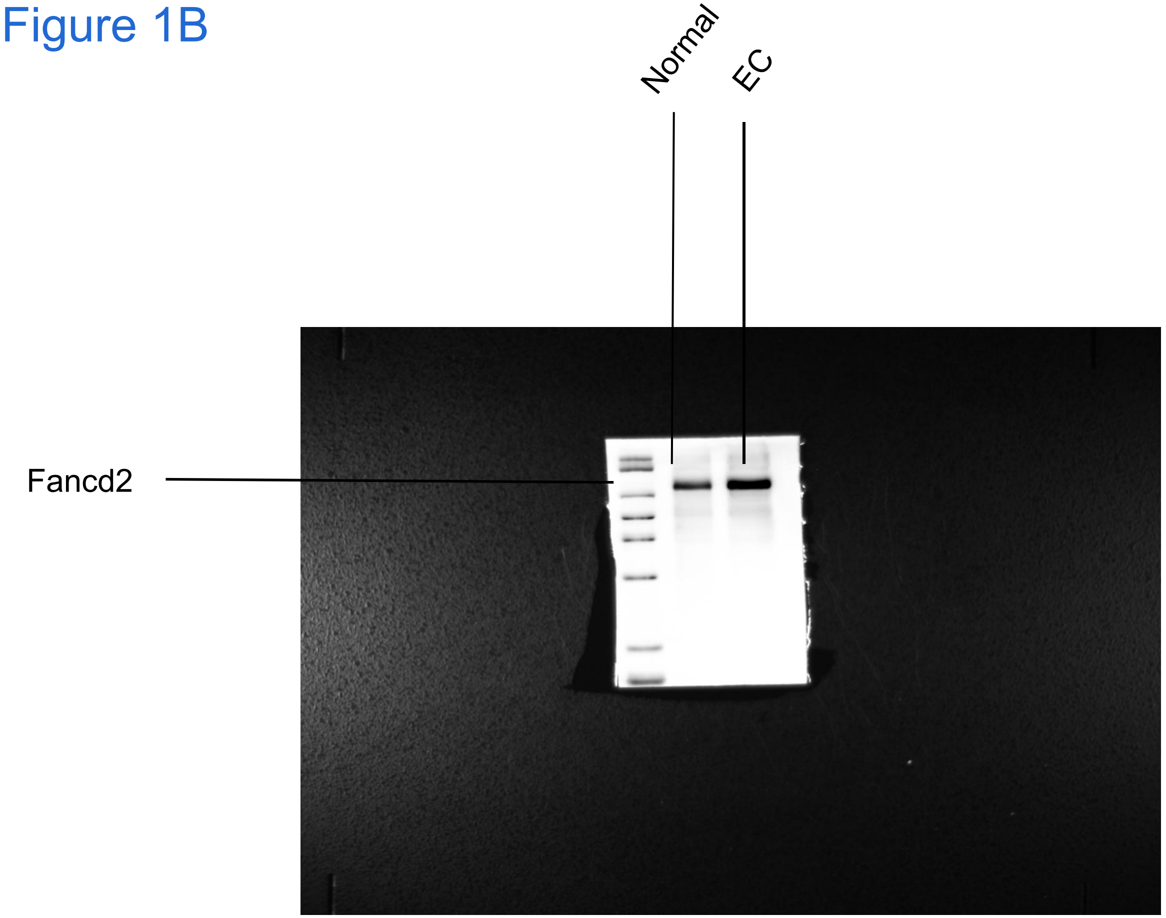

Figure 1B

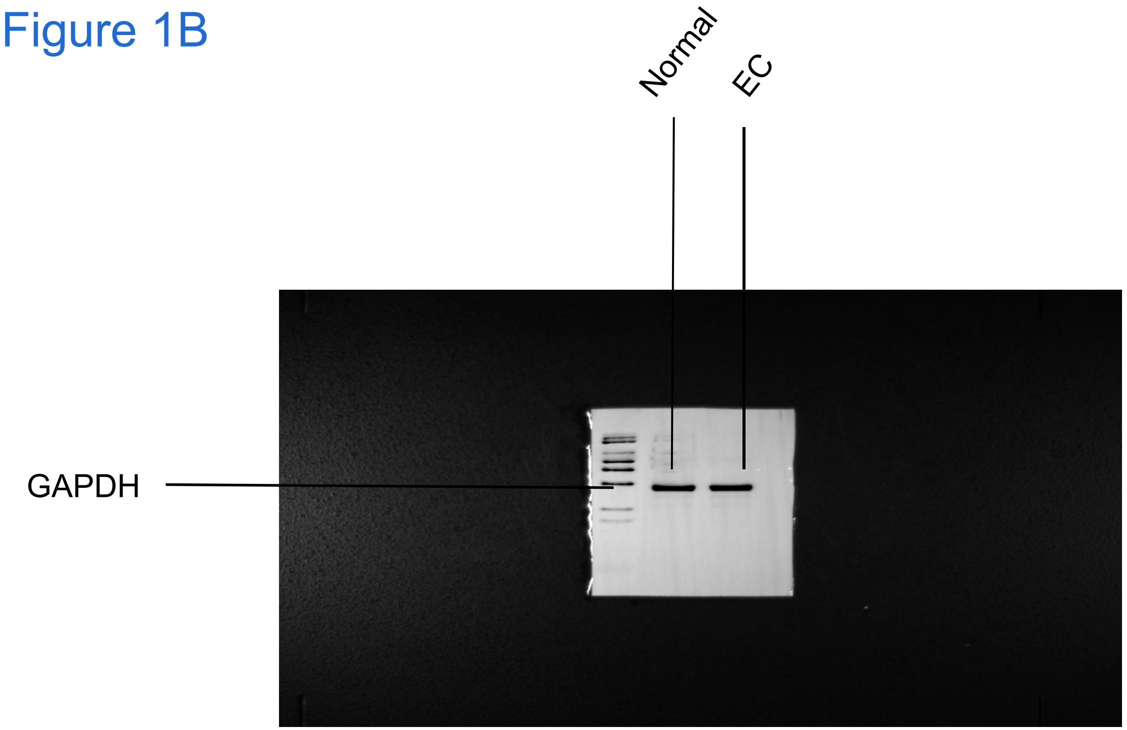

Figure 1D

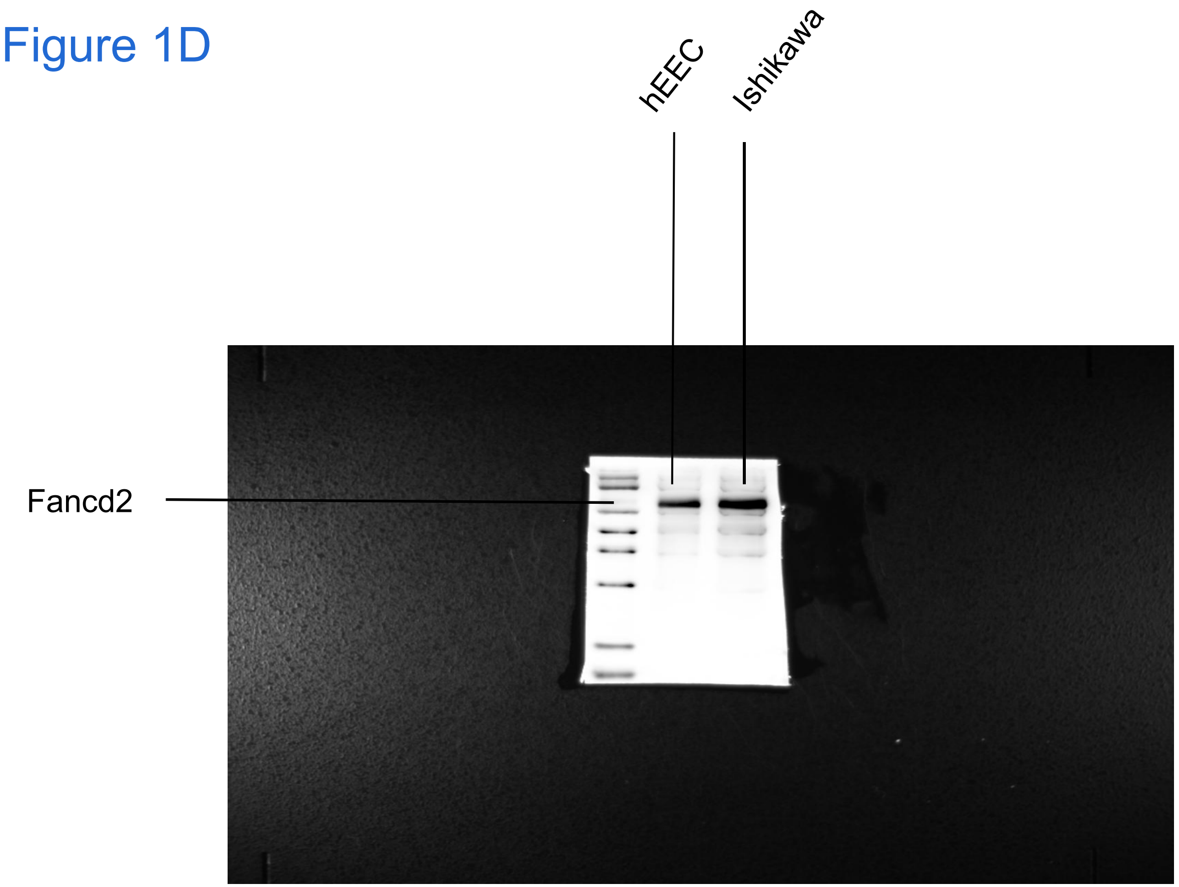

Figure 1D

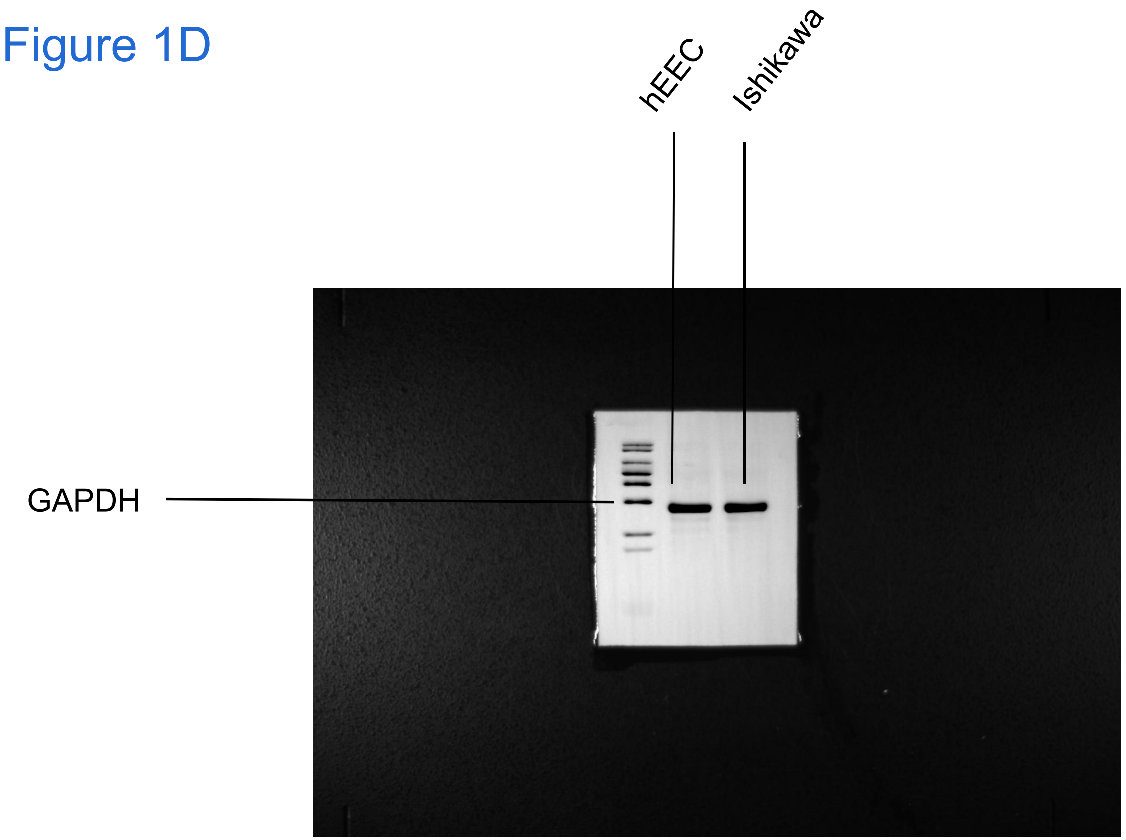

Figure 1F

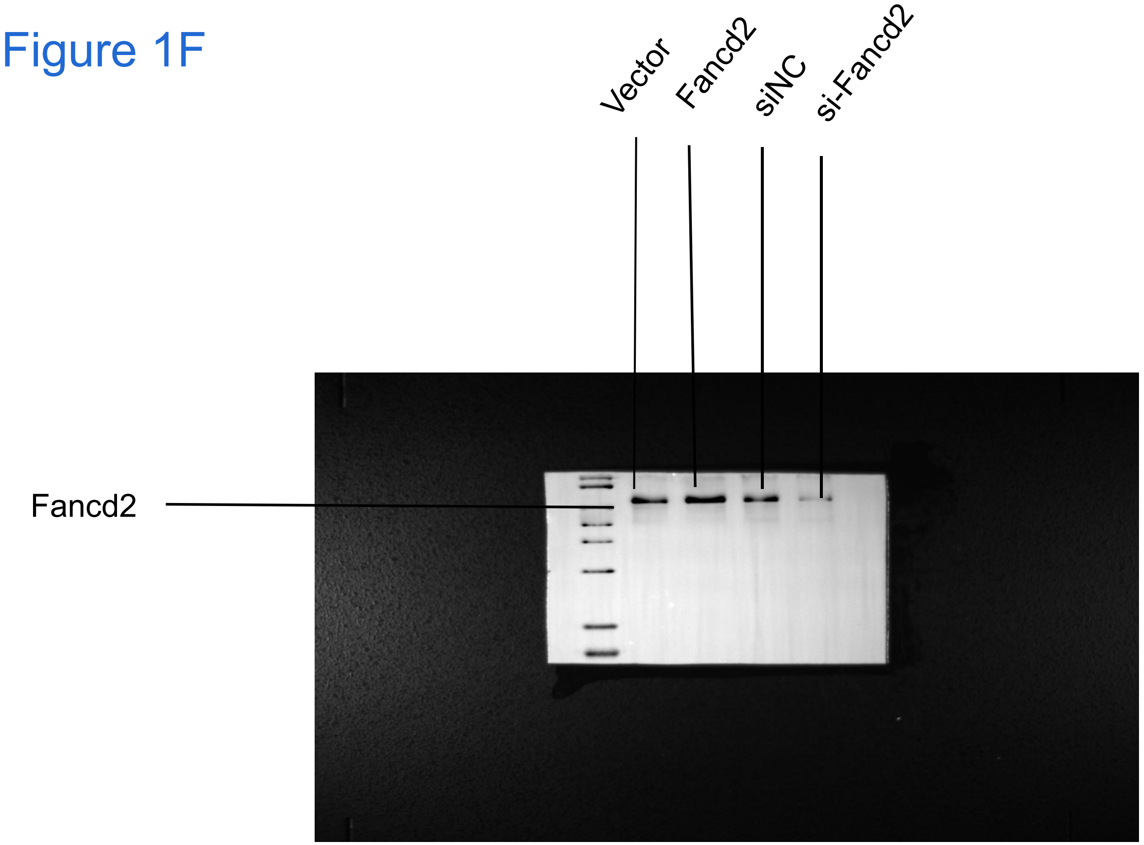

Figure 1F

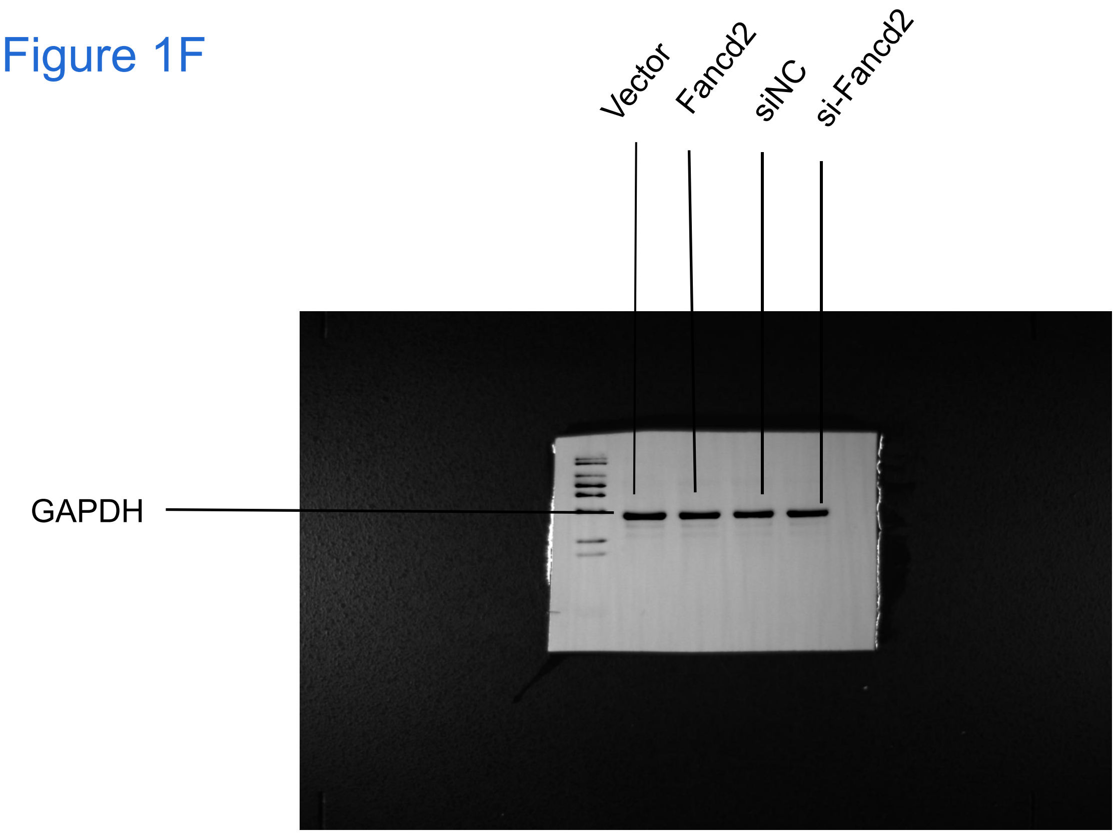

Figure 2B

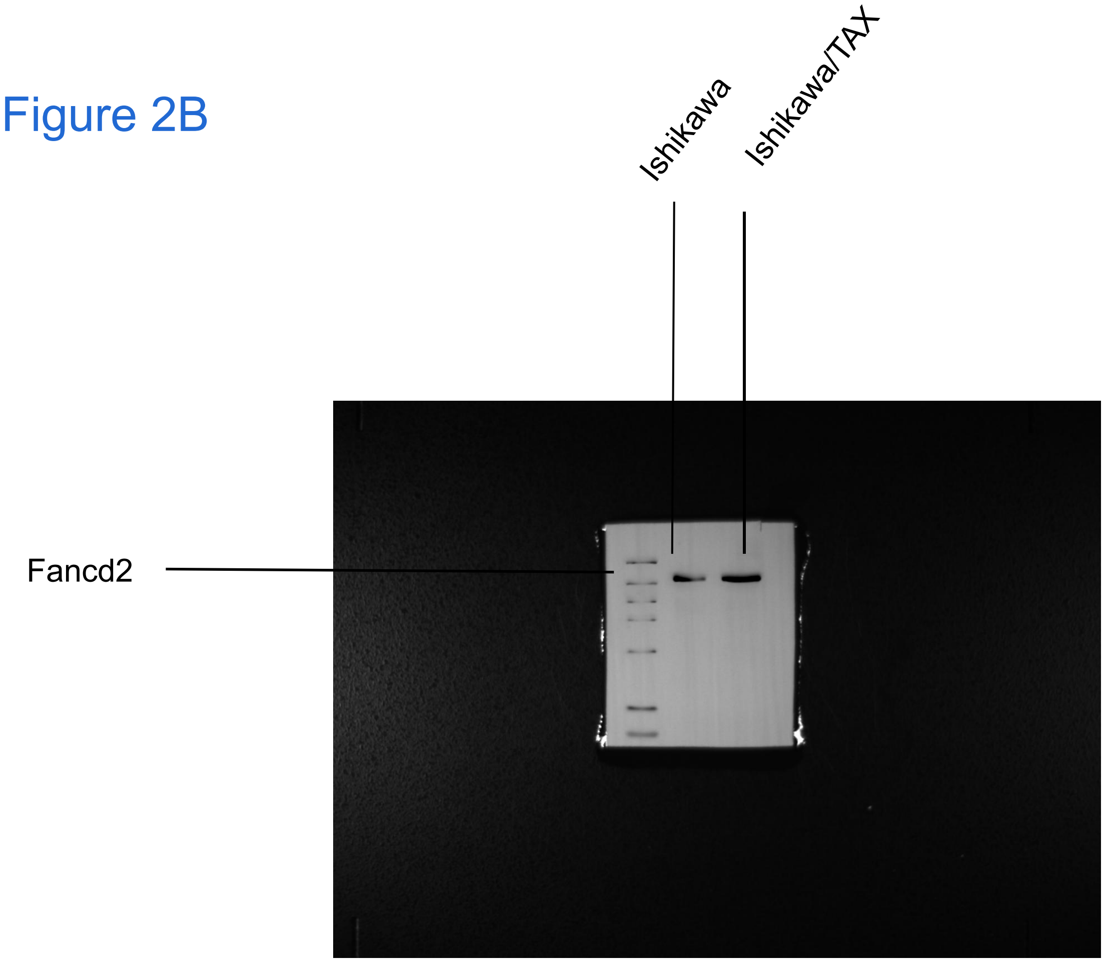

Figure 2B

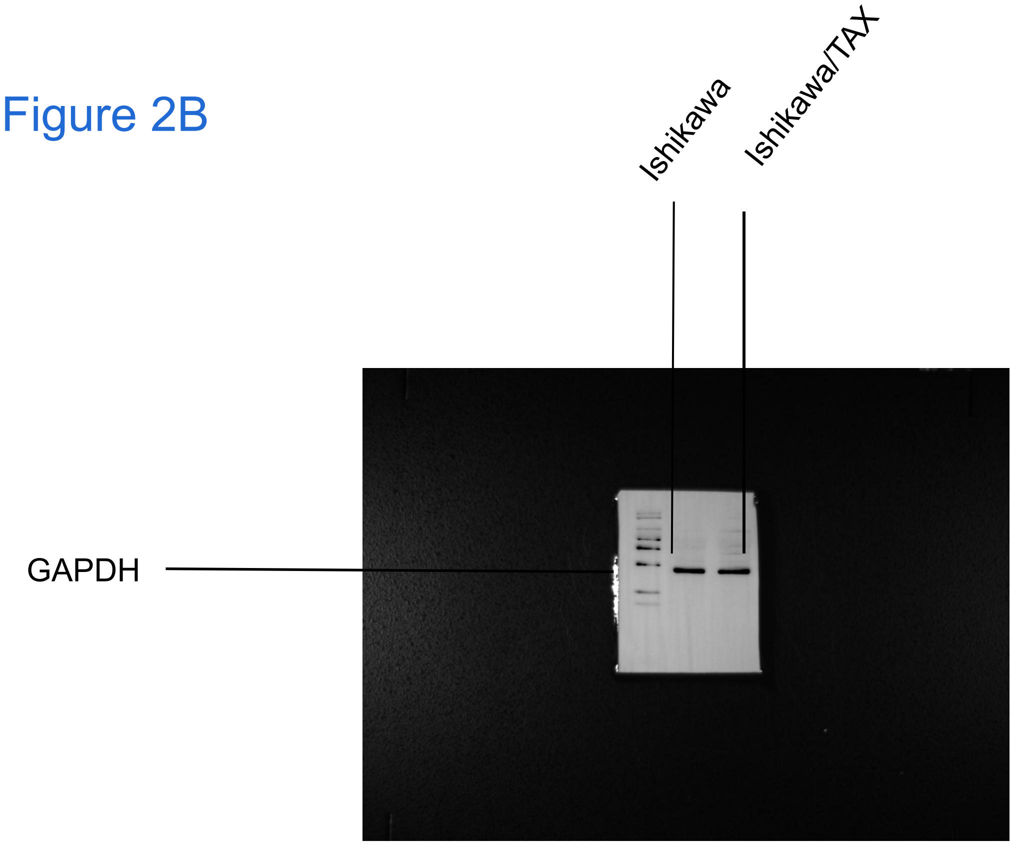

Figure 3E

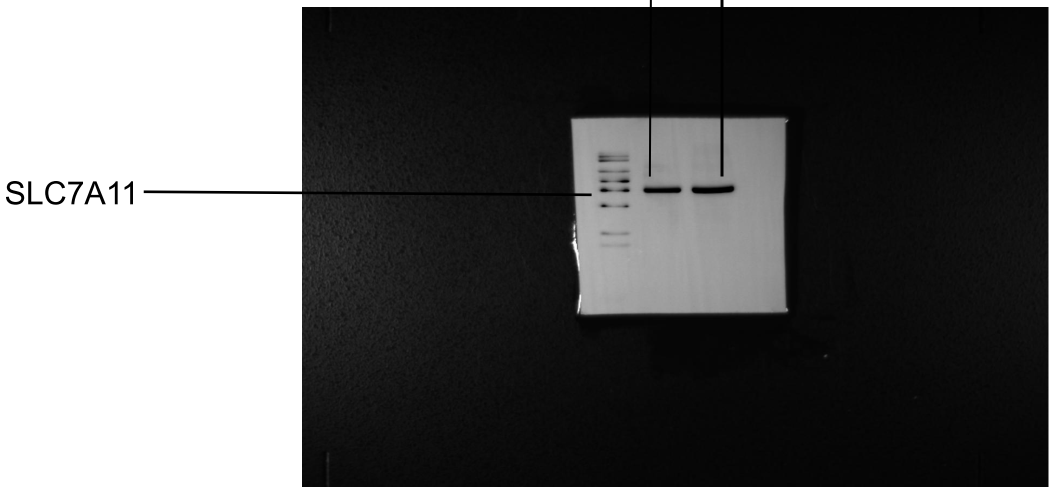

Figure 3E

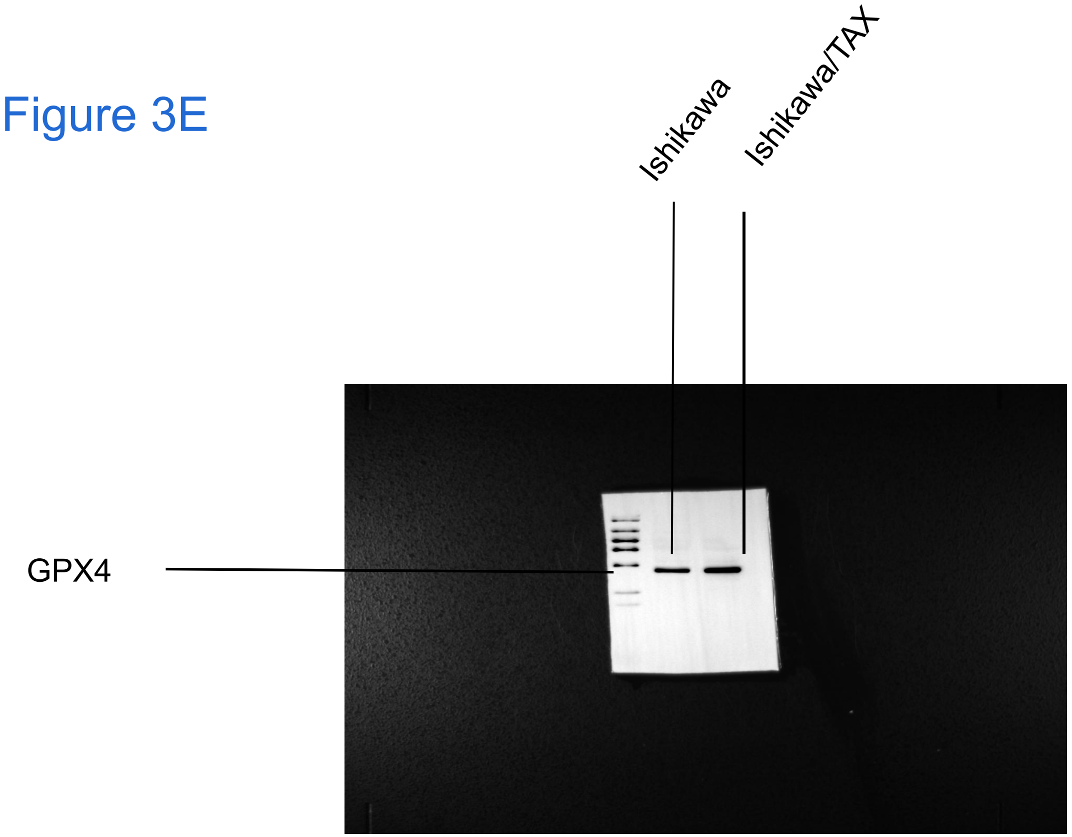

Figure 3E

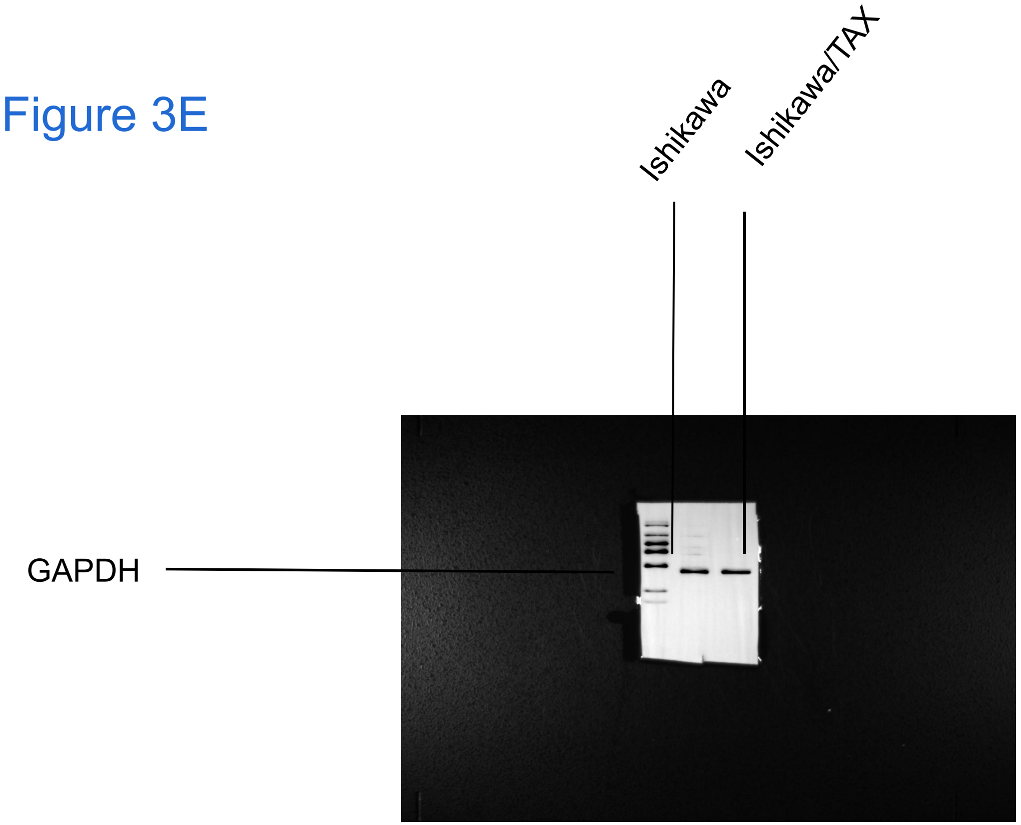

Figure 5F

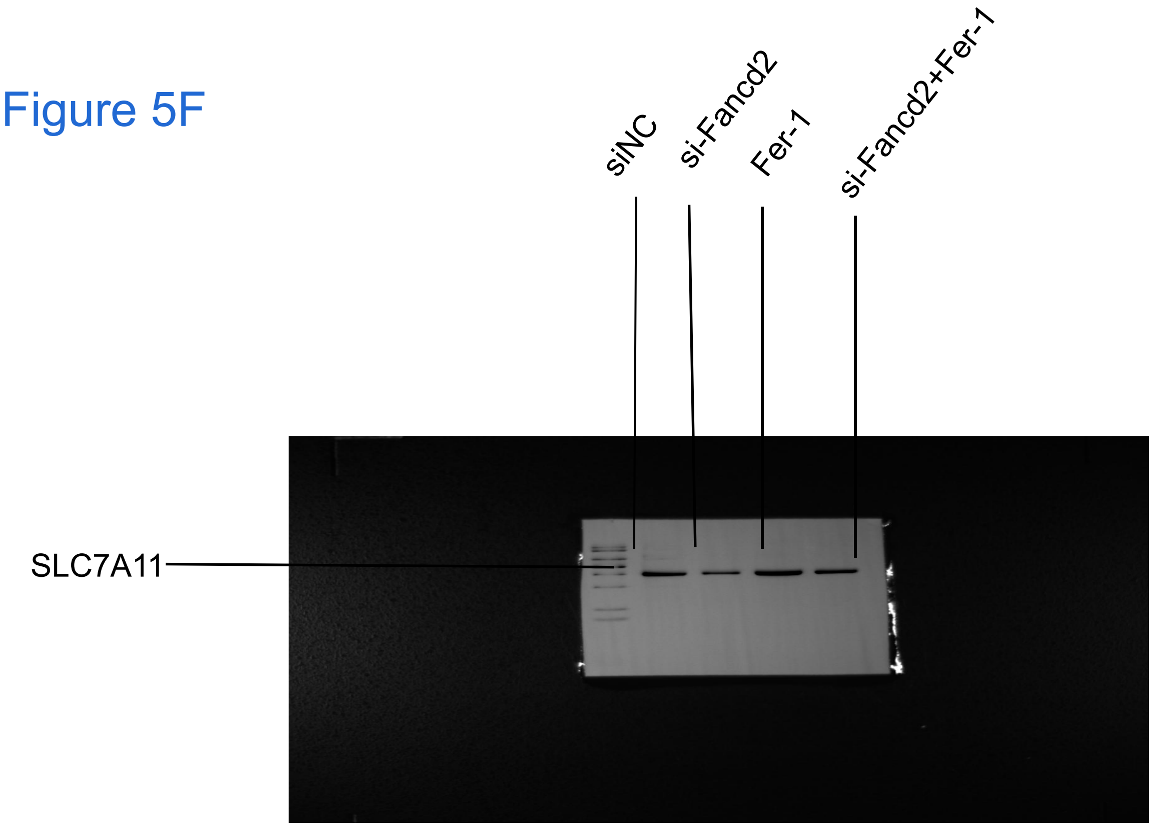

Figure 5F

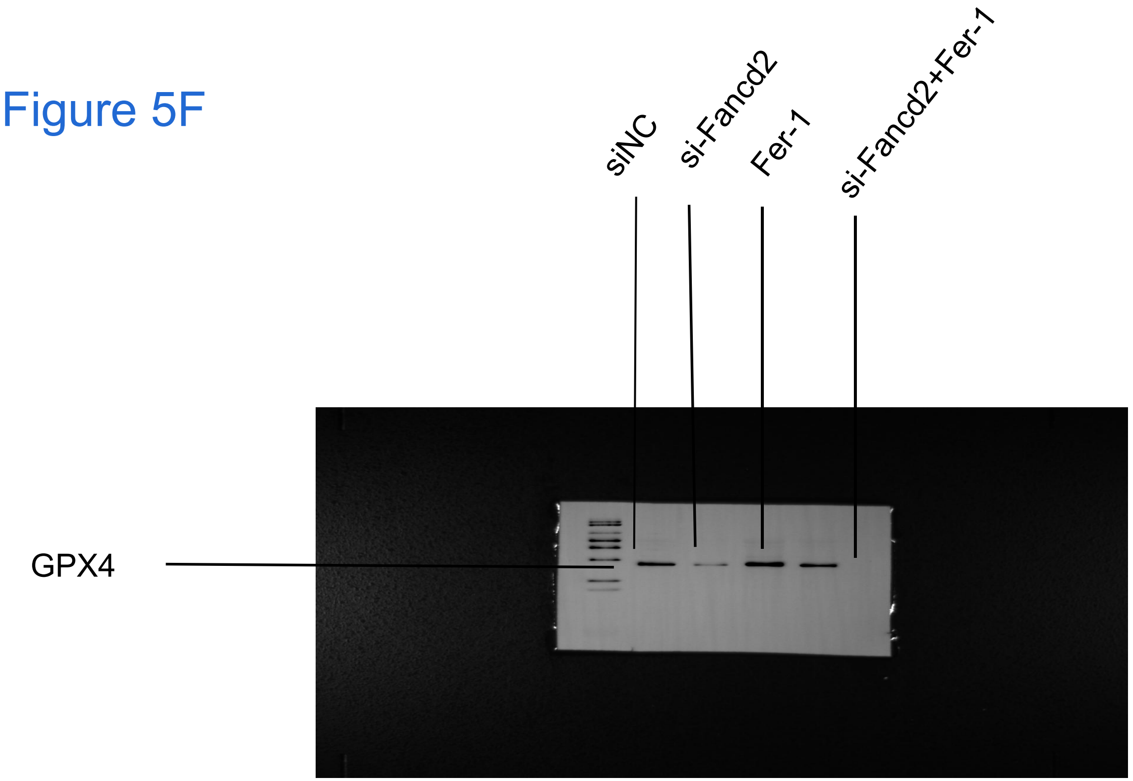

Figure 5F

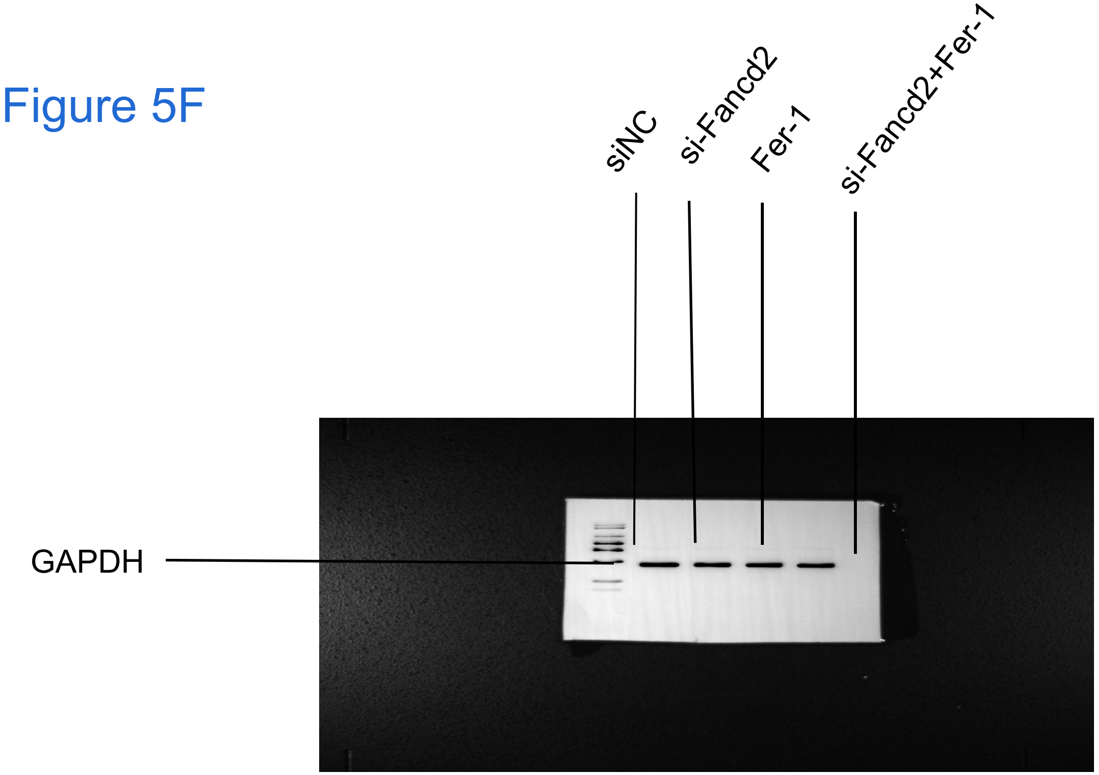

Supplement: Supplementary file 1 — Additional file 1. [file 12905_2023_2857_MOESM1_ESM.pdf]
